# Comparison of Toxic Metal Concentrations in Antidiabetic Herbal Preparations (ADHPs) Available in Bangladesh Using AAS and XRF Analytical Tools

**DOI:** 10.1155/2019/7154984

**Published:** 2019-12-19

**Authors:** Rausan Zamir, Nazmul Islam, Akhter Faruque

**Affiliations:** ^1^Department of Chemistry, University of Rajshahi, Rajshahi-6205, Bangladesh; ^2^Department of General Educational Development, Daffodil International University, Dhaka, Bangladesh

## Abstract

Widespread escalation of type 2 diabetes is a concern throughout the world. Developing countries are leading with patients suffering from diabetes-related complications. Plant-based therapeutic, antidiabetic herbal preparations (ADHPs) are being sought for long and the consumption is increasing in in Bangladesh. Plant-based antidiabetic preparations do not go through the screening procedure in terms of safety. Toxic metals in ADHPs have been investigated by two different methods: atomic absorption spectroscopy (AAS) and X-ray fluorescence (XRF). Then, metal concentrations obtained by AAS and XRF were compared. A total of eleven ADHPs were subjected to nondestructive XRF analysis and destructive AAS analysis. Results from the two methods were analyzed statistically by Pearson correlation coefficient (PCC), *r*_*xy*_. Pearson correlation coefficients were found to be −0.05, 0.94, and 1.00 for Mn, Cu, and Zn, respectively. Zn and Cu had significant strong positive correlation (*r*_*xy*_ = 1.00 and 0.94, respectively); however, very weak negative correlation was observed in Mn (*r*_*xy*_ = −0.05). The concentrations were regressed to observe the presence of linearity. Linear correlation was found for Zn and Cu which indicates a good agreement between AAS and XRF. However, very weak linear correlation in Mn indicates necessitating requirements for further investigation on getting scientific evidence of toxic metal assessment of the antidiabetic herbal preparations for searching and establishing instrumental agreement.

## 1. Introduction

Approach towards the use of herbal preparations has occupied two different ways. One way leads to traditionalism which depends on empirical appreciation of medicinal herbs. Use of medicinal plants and their preparations in treating disease are so long-standing that its inception is hardly found. At present, broad popularity of these products has increased few folds worldwide. It is estimated that 80% population of the developing world depend on herbal preparations as their primary healthcare [1, 2]. Nowadays, readiness of herbal preparations exceeded the drug stores and moved into the foodstuff retail shop outlets in superstores. A vast number of people are relying on herbal preparations not only for traditional belief but also for the eagerness of a fraction of them to know the science and clinical practice behind these types of preparations. And, this curiosity of people has opened door which depends on traditionalism and in that new approach, blending between science and traditionalism has become possible. This way is known as phytotherapy [3].

In developing countries, people lack health insurance and there is always shortage of money to treat diseases for poor to middle income people. Conventional drugs are available after visiting doctors who charge their consultancy, which in private clinic and hospitals is often burdensome. In addition, there is diagnosis cost. These all add up with the drug cost. On the other hand, doctors prescribing herbal preparations do not charge their consultancy fee, diagnosis is not suggested, and patients are required to pay money for preparations only. These incidents make herbal preparations cheap in comparison with allopathic drugs. Moreover, side effects of most modern drugs shifted a fraction of consumer's attention from orthodox ones to alternatives (herbal type) [4].

56.9 million deaths were recorded worldwide in 2016, where more than half (54%) were because of the top 10 causes. Diabetes is considered in the 6th place to be fatal among top ten causes of death [5]. An investigation conducted by Unicef showed that 1.6 million deaths were related to diabetes, and there was an increase of 0.6 million deaths from the year 2000 which accounted for 2.8% death worldwide in the year 2016. This is really a horrific account. Along with or in competition with allopathic drugs, scores of antidiabetic herbal preparations have penetrated the markets and drug stores. Electronic and print media play a role to attract the consumer towards herbal preparations [6, 7] for treating diabetes.

The origin being natural, herbal preparations are considered characteristically safe. But, investigation from scientific community does not support this traditional belief. There is evidence of toxicity and adverse effects of herbal preparation administration [8]. Herbal remedies are found to be contaminated with pesticides, microbes, heavy metals, and chemical toxins, leading to toxicity [9]. Both natural (geochemical characteristics of soil) and anthropogenic sources (contaminants in the soil, water, and air, and other during growth, transport, and storage conditions) are to be blamed for this occurrence. Metal toxicity in human beings, animals, and plants is not rare [10]. Metal toxicity may lead to dire consequences like malfunction and malformation of organs, abdominal pain, vomiting, severe anemia, and hemoglobulinuria with dark color stools. Apart from contamination, there is a possibility of intentional addition, i.e., adulteration which has been reported in Asian herbal preparation [8, 11].

An investigation into several toxic metals concentrations in ADHPs had been adopted by conventional analytical tool atomic absorption spectroscopy (AAS). AAS is destructive by its mechanism as the destruction of sample is done through digestion by acid solution [10]. Performing direct, in situ analysis of samples is not possible through AAS. Simultaneously, same herbal preparations were subjected to X-ray fluorescence (XRF) analysis for the toxic metals which were determined quantitatively by AAS. Performing XRF is direct and does not require digestion and hence nondestructive by its mechanism. Results from the two different methods were statistically analyzed to search any possible instrumental agreement. The statistical methods used in this study are correlation of association by Pearson correlation coefficient (PCC, *r*_*xy*_) and regression model.

## 2. Materials and Methods

### 2.1. Study Area and Sample Collection for AAS

**Figure 1:**
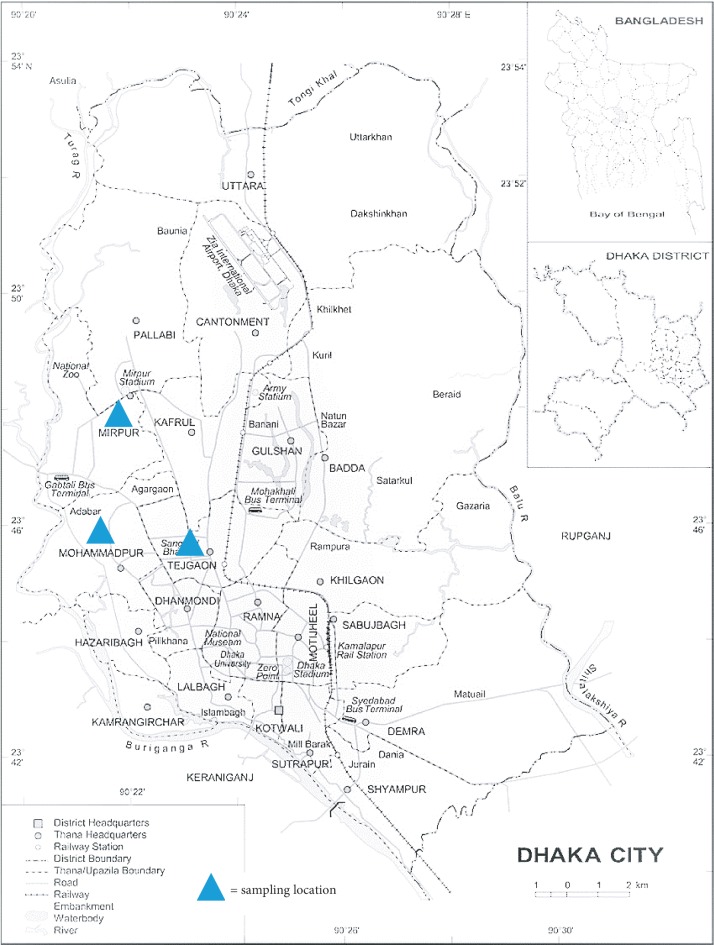
ADHPs sampling locations centered in Dhaka City, Bangladesh.

The study area was divided into three sampling zones (Mirpur 10, Mohammadpur, and Tejgaon) in Dhaka City, Bangladesh. A detailed map of sampling locations is shown in Figure 1. Sample 1–3 were collected from Mirpur 10, 4–7 were from Mohammadpur, and rest (8–11) were picked up from Tejgaon area (Table 1). Selection of the sampling zones from where the samples were collected was on the basis of herbal product selling hotspots, lower middle to poor people living in those densely populated areas and retailers selling herbal preparations as finished commercial pack. From there, the samples were transported to the laboratory and preserved as per the written direction.

After collection, the samples were placed onto individual porcelain dishes distinctly, and each dish with the particular sample was positioned in an oven. Temperature was maintained at around 70°C until a constant weight was attained. Using a mortar and pestle, oven-dried samples were pulverized to fine powder, followed by preservation in a plastic vial with the identification mark inside a desiccator. As ADHPs are made of organic materials, 1 g of homogeneous powder for herbal preparation was taken in a Teflon vessel, and initially, 10 mL HNO_3_ acid was used to decompose and abolish the organic materials. Next, an acid mixture of 6 mL conc. HNO_3_ (Merck, Germany), 3 mL conc. HClO_4_ (Merck, Germany), and 10 mL HF (Wako, Japan) was used for digestion of the samples. The prepared solution was evaporated to dryness on a ceramic hot plate (As One, Japan) (at 180°C) inside a fume hood on a hot plate. After that, the solid sample was dissolved in 5 mL of HF and 1 mL of HClO_4_ acid and heated to near dryness. After repetition of this procedure thrice, the dissolution was completed. Presence of HF was removed from the solution by addition of HNO_3_ acid and heated until white fumes were observed [12]. Finally, the residue was diluted to 0.1 N HNO_3_ and volume was made up to 25 mL in a PFA volumetric flask. For blank and standard reference materials (SRM 1753 a Tomato leaves, National Institute of Standards and Technology (NIST, Gaithersburg, Maryland, USA), the same procedures were performed [3].

### 2.2. Sample Analysis by AAS

Toxic metal ions were analyzed by an atomic absorption spectrophotometer (AAS 3110 Perkin-Elmer, Waltham, Massachusetts, USA) along with single-element hollow cathode lamps (AAS AA-7000, Shimadzu Corporation Japan) and a 10 cm air acetylene burner. The spectral band pass, the wavelengths, and other instrumental conditions were applied as prescribed by the manufacturer. The calibration curves for each element were prepared diluting the stock standard solution of 1000 mg/L (Wako Chemicals, Japan). Limit of detection (LOD) and limit of quantification (LOQ) for Pb, Zn, Cu, and Mn were 40 *μ*gm/L, 2.8 *μ*gm/L, 0.80 *μ*gm/L, 1.8 *μ*gm/L, and 120 *μ*gm/L and 8.4 *μ*gm/L, 2.4 *μ*gm/L, and 5.4 *μ*gm/L, respectively. This study was conducted in Bangladesh Scientific and Industrial Research (BCSIR, Dhaka, Bangladesh) [3].

### 2.3. Study Area and Sample Collection for XRF

Previously investigated (by AAS) eleven antidiabetic herbal preparations (ADHPs) (Table 1) were taken. Medicines were collected in air-tight plastic containers or glass bottles depending on their physical state, followed by the date of manufacturing, date of expiring, and batch number tabulation.

### 2.4. Sample Preparation and Analysis

Oven-dried (35°C for 5 minutes) samples were pulverized to a fine powder and pressed into a pellet of 13 mm size using a CARVER model manual pelletizing machine at 6–8 ton pressure. Pelletized samples were bombarded by the X-ray tube (25 V, 50 Micro A for 100 counts) after placing on the sample holder. Using solid-state Si-Li detector system, characteristic X-ray was detected. Through ADMCA and FP-CROSS software, the spectrum was analyzed.

### 2.5. Accuracy of Analytical Procedure

Considering the sensitivity of assessment, the accuracy and precision of the XRF analysis were checked by testing repeatedly the certified reference materials SRM 1571 (trace elements in Orchard leaves, National Bureau of Standards Certificate of Analysis, USA). Here, replication was 3 for all certified reference materials (Pb, Cu, Mn, and Zn), and the mean value is shown in Table 2. Replication was done on the same day; therefore, the precision was intraday. The results for the recoveries were between 90% and 103% (Table 2), which showed a good agreement between the measured and certified values.

## 3. Results and Discussion

Regulatory body (Jointly WHO/FAO) recommended permissible concentrations or maximum intake concentrations for Mn, Cu, Zn, and Pb in herbal preparations which are 320 mg/kg, 3 mg/kg, 50 mg/kg, and 10 mg/kg, respectively [13, 14]. All instances of Cu concentrations determined by XRF and AAS in the supplied antidiabetic herbal preparations (ADHPs) (Table 3) exceeded recommended permissible concentrations set by the WHO/FAO. Excessive intake of copper is linked with dermatitis and irritation of the upper respiratory tract, abdominal pain, nausea, diarrhea, vomiting, and liver damage [15, 16]. However, no instances for Mn determined by AAS and XRF passed safe limits set by the FAO/WHO (Table 3). Very few instances ADHP-7 by XRF and ADHP-1 and ADHP-7 by AAS were recorded with surpassing values of safe limits fixed by the regulatory body for Zn. Zn is a must-needed element for proper growth and helps in blood clotting, thyroid functioning, and protein and DNA syntheses. However, if zinc uptake exceeds permissible limits, it yields toxic effects. The immune system and blood lipoprotein levels are affected by the toxic effects produced by Zn [17]. Only ADHP-7 assessed by AAS for Pb crossed recommended permissible concentrations provided by the WHO and FAO (Table 3). Events of neurotoxicity and nephrotoxicity and many others adverse health effects are associated with Pb toxicity [18].

Pearson correlation coefficient (PCC) is denoted by *r*_*xy*_ and referred to as the sample correlation coefficient or the sample Pearson correlation coefficient (SPSS) [19]. For a given paired data set {(*x*_1_, *y*_1_),…,  (*x*_*i*_, *y*_*i*_)}  consisting of *n* pairs, it can be formulated as follows:

(1) $$\begin{array}{c} r_{xy}=\frac{\sum_{i=1}^{n}\left(xi-\bar{x}\right)\left(yi-\bar{y}\right)}{\sqrt{\sum_{i=1}^{n}{\left(xi-\bar{x}\right)}^{2\;}}\sqrt{\sum_{i=1}^{n}{\left(yi-\bar{y}\right)}^{2\;}}}, \end{array}$$

where *n* is the sample size, *xi* and *yi* are the individual sample points indexed with *i*, and $\bar{x}$ = (1/*n*)∑_*i*=1_^*n*^*xi* is the sample mean and analogous for $\bar{y}$.

According to the Cauchy–Schwarz inequality, PCC valued between +1 and −1. PCC value 1 indicates positive linear correlation, 0 is no linear correlation, and −1 is total negative linear correlation. Using equation (1), Pearson correlation coefficient for concentration sets of Mn, Cu, and Zn was determined (Table 4) as follows.

  Set 1 for Zn:

(2) $$\begin{array}{c} f\left(x_{i},y_{i}\right)=\left\{\left(8,59.3\right),\left(6.6,NF\right),\left(11,48\right),\left(6.6,2\right),\left(9.4,5.38\right),\left(5.8,3.75\right),\left(181.4,1937\right),\left(9.6,2.9\right),\left(7.6,2.4\right),\left(6.4,NF\right),\left(13.8,3.75\right)\right\}. \end{array}$$

  Set 2 for Cu:

(3) $$\begin{array}{c} \;f\left(x_{i},y_{i}\right)=\left\{\left(7.4,5.46\right),\left(6.6,NF\right),\;\left(8.8,6.12\right),\left(8.6,7.25\right),\left(11.2,5.38\right),\left(7.2,23.2\right),\left(19.6,49.6\right),\left(8.2,8.9\right),\left(7.8,4.5\right),\left(7.4,4.25\right),\left(7.8,10\right)\right\}. \end{array}$$

  Set 3 for Mn:

(4) $$\begin{array}{c} f\left(x_{i},y_{i}\right)=\left\{\left(72,211\right),\left(248,32.4\right)\left(160,108\right),\left(140,163\right)\left(86,75\right),\left(72,23.2\right),\left(124,148\right),\;\left(70,15.2\right),\left(74,18.3\right),\left(84,4.63\right),\left(180,9.25\right)\right\}. \end{array}$$

Set 1 represents values for Zn determined by XRF and AAS in sets. In set 1, Pearson correlation coefficient was found to be 1. Here, confirmation of an excellent performance was observed over a range of concentrations. As *r* results, both instruments (XRF and AAS) showed an excellent agreement while considering Pearson correlation coefficient (PCC),  *r*_*xy*_  (Table 4). In case of set 2 for Cu, PCC, *r*_*xy*_ value was found 0.94. This value is also indicating a strong positive agreement between results from AAS and XRF instruments over a range of concentrations. In the literature, a very good correlation between the flame AAS and the XRF techniques was found by Clark [20]. However, PCC, *r*_*xy*_ value, for Cu in set 3 showed a value of −0.05 which indicates a very weak negative correlation. As a result, the correlation of association between XRF and AAS was not established with this value. Here, instrument agreement fails to correlate.

**Figure 2:**
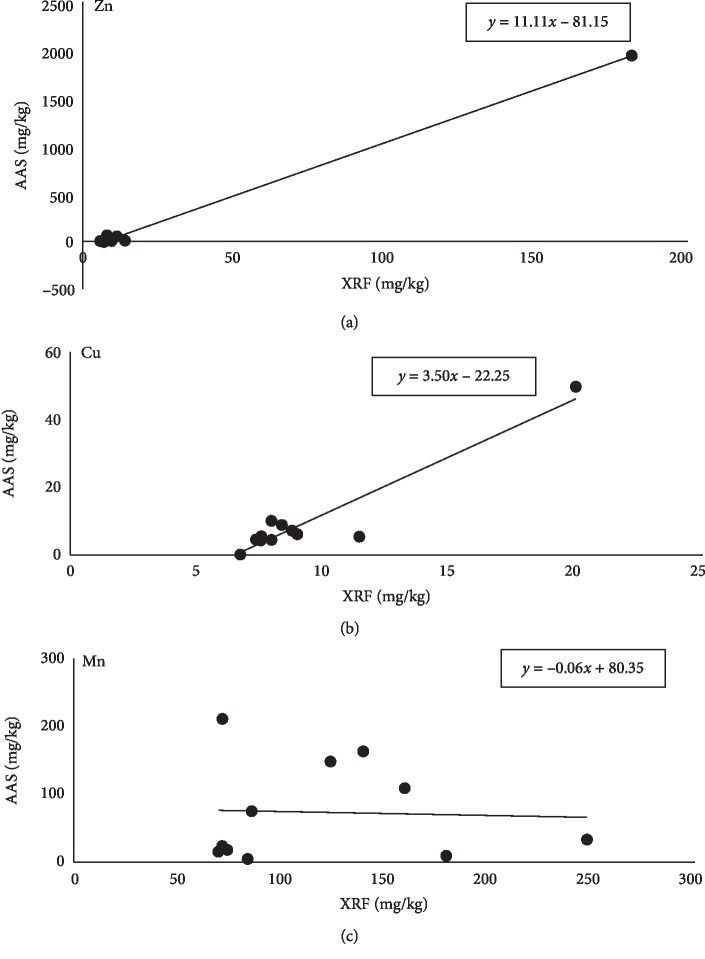
Regression line drawn from the correlation between Zn, Cu, and Mn measurements obtained by XRF and AAS.

Correlation between metal concentrations was regressed to a linear model with slope, *m*, and intercept, _*c*_, as a function of concentration. The values of *m*, *c*, and other regression statistics are shown in Table 5. The most common method for fitting a regression line is the method of least-squares which calculates the best-fitting line for the observed data by minimizing the sum of the squares of the vertical deviations (residuals) from each data point to the line. No cancellations between positive and negative values are seen in this method, because the residuals are first squared, then summed, and plotted (Figure 2).

Squared residuals, *Q*, can be written as

(5) $$\begin{array}{c} Q=\;\underset{i}{\sum }r_{i}^{2}=\underset{i}{\sum }{\left(y_{i}-\hat{y_{i}}\right)}^{2}=\underset{i}{\sum }{\left(y_{i}-mx_{i}-c\right)}^{2}, \end{array}$$

where *r*_*i*_  is the residual, *y*_*i*_ is the experimental point at the *y* axis, $\hat{y_{i}}$ is the fitted point = *mx*_*i*_+*c*, *m* is the slope, and *c*  is the intercept.

The mathematical relationship describing the linear relationship between concentrations for each metals determined by XRF and AAS is called the regression model, which has the following mathematical representation:

(6) $$\begin{array}{c} y=mx+c. \end{array}$$

Calculating slope, *m*, and intercept, *c* for each sets of statistical data (Table 5), the regression model can be represented for individual metals Zn, Cu, and Mn by the following equations (7a)–(7c), respectively:

(7a) $$\begin{array}{c} y=11.11x-81.15, \end{array}$$

(7b) $$\begin{array}{c} y=-3.50x-22.25, \end{array}$$

(7c) $$\begin{array}{c} y=-0.06x+80.35. \end{array}$$

From the regression line, excellent linearity was found for Zn and Cu and very poor linearity was seen for Mn assessed by XRF and AAS analytical tool (Figure 2). Another investigation conducted by Radu and Diamond [21] which was about assessment of toxic metals from soil samples leading to comparison between AAS and XRF also showed similar types of regression lines. These findings also support our values depicted by Pearson correlation coefficient, PCC, *r*_*xy*_.

**Figure 3:**
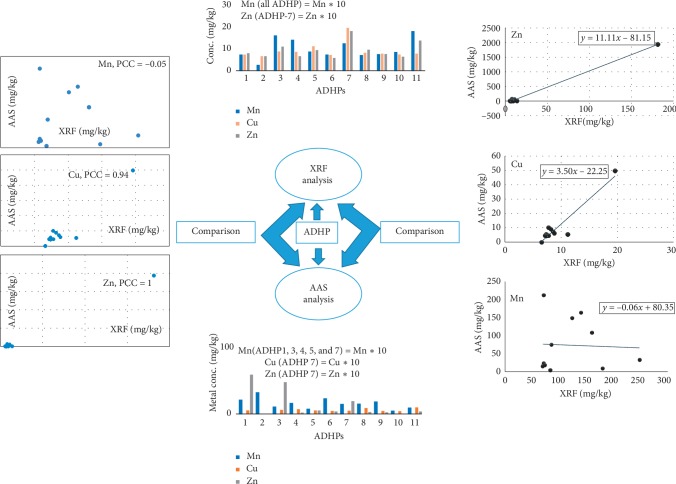
Schematic representation for investigating instrument agreement.

The whole discussion can be summarized using the schematic representation shown in Figure 3. This figure reveals that concentrations of Cu and Zn by both instruments (AAS and XRF) showed good agreement while assessed by Pearson correlation coefficient (shadowed left side of Figure 3). Again, the regression models for Cu and Zn were found with significant high slope, indicating a good agreement between AAS and XRF analyses (shadowed right side of Figure 3).

## 4. Conclusion

Considering public health safety metal concentrations in ADHPs had been determined by AAS and XRF where destructive AAS assessment was performed before nondestructive XRF analysis. Using Pearson correlation coefficient (PCC), linear relationship between two different assessments was searched and the relationship was regressed to a model. Strong linear correlation was observed in Zn and Cu (*r*_*xy*_ = 1.00 and 0.94, respectively). This result is the opening possibility of flexibility in instrument selection for determination of metal concentration in herbal preparations and in future possible transformation to portable device if results are recurring. However, very poor linear relationship for Mn (*r*_*xy*_ = −0.05) indicates significant variation in results obtained by two different methods. In both instances, there is an indication for more investigation for receiving reproducibility of scientific data in toxic metal assessment of the antidiabetic herbal preparations.

## Figures and Tables

**Table 1:** List of antidiabetic herbal preparations (ADHPs) under investigation.

S no.	Code	Dosage form	Dosage	Weight per tablet or capsule (mg)
1	ADHP-01	Capsule	1-2 capsule, 1-2 times	620
2	ADHP-02	Capsule	2 capsule, 2 times	500
3	ADHP-03	Capsule	1 capsule, 2 times	490
4	ADHP-04	Capsule	1-2 capsule, 2 times	510
5	ADHP-05	Capsule	1 capsule, 2-3 times	505
6	ADHP-06	Tablet	1-2 tablet, 2-3 times	500
7	ADHP-07	Tablet	1-2 tablet, 2 times	560
8	ADHP-08	Tablet	10 gms, 2-3 times	450
9	ADHP-09	Tablet	1-2 tablets, 2 times	550
10	ADHP-10	Tablet	1-2 tablets, 3 times	3000
11	ADHP-11	Capsule	1 capsule, 2 times	520

**Table 2:** Analytical results obtained on certified reference materials (*μ*g/g).

Element	Certified value	Mean measured value (*n* = 3, intraday)	Recovery (%)	Accuracy (%)
Certified reference material	Analytical results			
Pb	45	43.02	95.60	−4.40
Mn	91	85.28	93.71	−6.29
Cu	12	10.87	90.58	−9.42
Zn	25	25.8	103.20	3.20

Statistical analysis of data was performed using Microsoft Excel 2016 Data Analysis Tool-Pack. Pearson correlation coefficient was determined, and the regression model was developed for each set of toxic metals assessed by the instruments (AAS and XRF).

**Table 3:** Toxic metal concentrations by XRF and AAS.

Sl no.	Code	XRF (mg/kg)				AAS (mg/kg)			
		Mn	Cu	Zn	Pb	Mn	Cu	Zn	Pb
1	ADHP-01	72	7.4	8	<LOQ	211	5.46	59.3	0.45
2	ADHP-02	248	6.6	6.6	<LOQ	32.4	NF	NF	NF
3	ADHP-03	160	8.8	11	<LOQ	108	6.12	48	0.75
4	ADHP-04	140	8.6	6.6	<LOQ	163	7.25	2	9.88
5	ADHP-05	86	11.2	9.4	<LOQ	75	5.38	5.38	8.5
6	ADHP-06	72	7.2	5.8	<LOQ	23.2	4.5	3.75	13.38
7	ADHP-07	124	19.6	181.4	<LOQ	148	49.6	1937	539
8	ADHP-08	70	8.2	9.6	<LOQ	15.2	8.9	2.9	3.9
9	ADHP-09	74	7.8	7.6	<LOQ	18.3	4.5	2.4	5.75
10	ADHP-10	84	7.4	6.4	<LOQ	4.63	4.25	NF	9.38
11	ADHP-11	180	7.8	13.8	<LOQ	9.25	10	3.75	8.5

NF = not found; LOQ = limit of quantification.

**Table 4:** Pearson correlation coefficient for toxic metals assessed by AAS and XRF analytical tools.

	Mn	Cu	Zn	Pb
PCC, *r*_*xy*_	−0.05	0.94	1	NF

**Table 5:** Experimental metal concentrations and statistical parameters for the linear regression model.

Toxic metal	Coordinates (*x*_*i*_, *y*_*i*_)	Data point, *n*	Intercept, *c*	Slope, *m*	*R* ^2^
Zn	$\begin{array}{c} \left(8,59.3\right),\left(6.6,NF\right),\left(11,48\right),\\ \left(6.6,2\right),\left(9.4,5.38\right),\left(5.8,3.75\right),\\ \left(181.4,1937\right),\left(9.6,2.9\right),\left(7.6,2.4\right),\\ \left(6.4,NF\right),\left(13.8,3.75\right) \end{array}$	11	−81.15	11.11	1.0
Cu	$\begin{array}{c} \left(7.4,5.46\right),\left(6.6,NF\right),\;\left(8.8,6.12\right),\\ \left(8.6,7.25\right),\left(11.2,5.38\right),\left(7.2,23.2\right),\\ \left(19.6,49.6\right),\left(8.2,8.9\right),\left(7.8,4.5\right),\\ \left(7.4,4.25\right),\;\left(7.8,10\right) \end{array}$	11	−22.25	3.48	0.9
Mn	$\begin{array}{c} \left(72,211\right),\left(248,32.4\right)\left(160,108\right),\\ \left(140,163\right)\left(86,75\right),\left(72,23.2\right),\\ \left(124,148\right),\;\left(70,15.2\right),\left(74,18.3\right),\\ \left(84,4.63\right)\left(180,9.25\right) \end{array}$	11	80.35	−0.06	0.0

## Data Availability

The data used to support the findings of this study are available in tables attached, and if needed, raw data further would be provided from the corresponding author upon request.
